# Development of a top-down MS assay for specific identification of human periostin isoforms

**DOI:** 10.3389/fmolb.2024.1399225

**Published:** 2024-06-19

**Authors:** Christian E. Rusbjerg-Weberskov, Megan S. Gant, Julia Chamot-Rooke, Nadia Sukusu Nielsen, Jan J. Enghild

**Affiliations:** ^1^ Department of Molecular Biology and Genetics, Aarhus University, Aarhus, Denmark; ^2^ Mass Spectrometry for Biology, Institut Pasteur, Université Paris Cité, CNRS UAR 2024, Paris, France

**Keywords:** top-down mass spectrometry, periostin, parallel reaction monitoring, chemical cleavage, method development

## Abstract

Periostin is a matricellular protein encoded by the POSTN gene that is alternatively spliced to produce ten different periostin isoforms with molecular weights ranging from 78 to 91 kDa. It is known to promote fibrillogenesis, organize the extracellular matrix, and bind integrin-receptors to induce cell signaling. As well as being a key component of the wound healing process, it is also known to participate in the pathogenesis of different diseases including atopic dermatitis, asthma, and cancer. In both health and disease, the functions of the different periostin isoforms are largely unknown. The ability to precisely determine the isoform profile of a given human sample is fundamental for characterizing their functional significance. Identification of periostin isoforms is most often carried out at the transcriptional level using RT-PCR based approaches, but due to high sequence homogeneity, identification on the protein level has always been challenging. Top-down proteomics, where whole proteins are measured by mass spectrometry, offers a fast and reliable method for isoform identification. Here we present a fully developed top-down mass spectrometry assay for the characterization of periostin splice isoforms at the protein level.

## 1 Introduction

**FIGURE 1:**
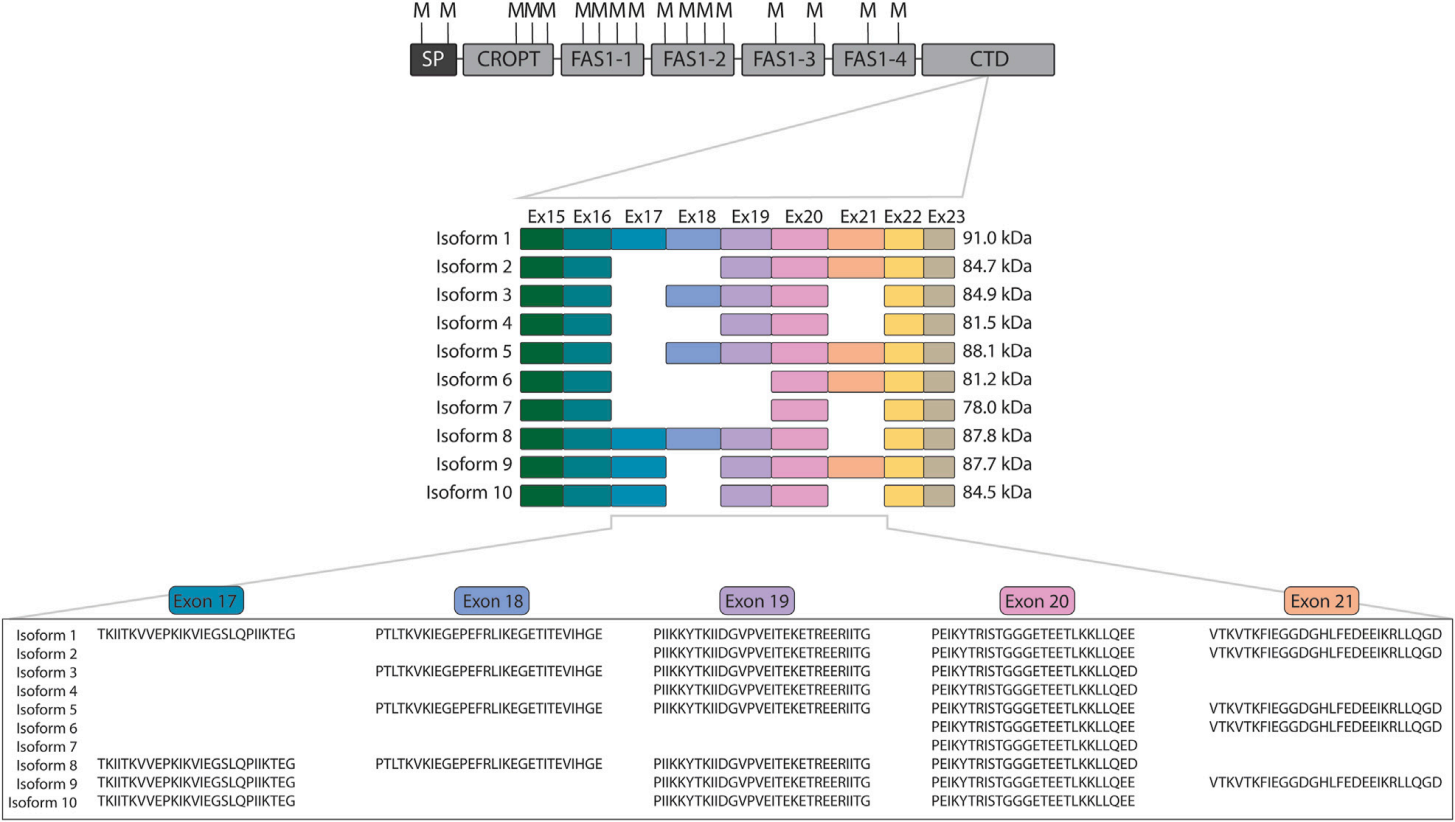
Overview of periostin domain composition and isoforms. The domain composition of periostin is shown with the location of all methionine residues indicated above (M). The cleavage at methionine residues using CNBr releases the intact variable C-terminal domain for all isoforms. The exons encoding each periostin isoform are displayed below together with the mature molecular weight of the isoforms after signal peptide removal. Indicated below are the protein sequences of the alternatively spliced exons. SP: Signal peptide, CROPT: Cysteine-rich domain of periostin and TGFBIp, FAS1: Fasciclin 1, CTD: C-terminal domain.

In the very first investigation of the POSTN gene encoding periostin, then named osteoblast-specific factor 2, it was observed to be subject to alternative splicing (Takeshita et al., 1993). Since then, studies on periostin have revealed the existence of ten splice isoforms of periostin (Castronovo et al., 2006; Morra et al., 2011; Morra et al., 2012) of which isoforms 9 (Morra et al., 2011) and 10 (Morra et al., 2012) are often overlooked. Periostin is composed of an N-terminal cysteine-rich domain of periostin and TGFBIp (CROPT) domain, four fasciclin-1 domains, and a C-terminal domain that harbors the variation occurring from alternative splicing (Figure 1). Variation occurs from splicing of the similarly sized exons 17, 18, 19, and 21 that encode part of the C-terminal domain (Figure 1). The C-terminal domain of periostin is disordered (Rusbjerg-Weberskov et al., 2023) and constitute 11%–26% of the protein depending on the isoform.

Periostin is a matricellular protein expressed in most connective tissue (Gillan et al., 2002; Tilman et al., 2007). Here it facilitates fibrillogenesis, organization of the extracellular matrix (Norris et al., 2007; Maruhashi et al., 2010), and induces cell signaling by anchoring to integrin receptors on cell surfaces (Gillan et al., 2002; Masuoka et al., 2012). Periostin is involved in various physiological processes and diseases including wound healing (Elliott et al., 2012; Ontsuka et al., 2012), development of bone and teeth (Rios et al., 2005), atopic dermatitis (Masuoka et al., 2012; Mitamura et al., 2018), and cancer (Tilman et al., 2007; Mikheev et al., 2015). However, the role of the individual isoforms in these processes remains largely unknown (reviewed recently by Kudo et al. (Kudo, 2017)). Alternative splicing is observed to take place in a regulated manner. It differs between tissues, during development, or in disease (Takayama et al., 2006; Zhu et al., 2008; Morra et al., 2011; Morra et al., 2012; Nance et al., 2014; Cai et al., 2019) and this is a strong indicator that the different isoforms have distinct functionalities. For example, isoform 1 and 3 but not isoform 4 decrease metastasizing of tumor cells in an *in vivo* mouse model of lung metastasis (Kim et al., 2008), and isoform 3 but not isoforms 2 or 4 plays a role in TGF-β signaling in human retinal epithelial cells (Yoshida et al., 2011). Specific and sensitive assays are essential for understanding the functions of different periostin isoforms. RT-PCR is the most widespread method used to study expression of splice isoforms (Takayama et al., 2006; Kim et al., 2008; Bai et al., 2010; Morra et al., 2011; Morra et al., 2012; Cai et al., 2019), but an analysis at the proteoform level would be superior as it more accurately describes the actual isoform profile. Mass Spectrometry (MS)-based proteomics at the peptide level (bottom-up proteomics or BUP) is the most widely used MS-based technique for protein detection and characterization. A major drawback of this approach is that it is not possible to distinguish between proteoforms (Smith and Kelleher, 2013). Proteoforms are different forms of the same protein (same gene) that share large portions of the same amino acid sequence, which in our case are periostin isoforms. In BUP experiments, when several proteoforms are trypsin digested, they produce many shared peptides that can be used to identify the protein but the proteoform information is lost in the digestion. In contrast, by analyzing intact proteins or large protein fragments containing the sites of variation using top-down proteomics (TDP) it is possible to distinguish between similar proteoforms. In the TDP workflow, intact proteins or large protein fragments are separated by liquid chromatography and introduced directly into the high resolution mass spectrometer to generate MS and MS/MS spectra that can be used for proteoform sequencing (Chen et al., 2018). TDP is not as well established as the commonly used BUP, because intact proteins are challenging to analyze. Large molecules do not ionize or fragment as well as peptides and are consequently less likely to be “seen” by the MS. Therefore, tailored TDP methods as the one presented here are needed in the use of TDP to distinguish highly similar proteoforms. Two studies by Dupré et al. demonstrate how TDP can be used both to identify proteomes in complex samples, such as bacteria lysates in a data-dependent acquisition mode, and to sequence-specific proteins of interest in targeted analyses (Dupré et al., 2021a; Dupré et al., 2021b). By combining these approaches utilizing online liquid chromatography to handle complex samples and use targeting of the isoforms of interest, TDP will be a powerful tool in isoform discovery. Intact periostin splice isoforms are unsuitable for most TDP experiments because they are too large (78–91 kDa). To overcome this, the region of interest (the CTD region) needs to be cleaved off and analyzed separately. The use of chemical cleavage to generate large fragments suitable for TDP analysis has been reported (Srzentić et al., 2018). By removing the N-terminal part of periostin by chemical cleavage on the C-terminal side of methionine residues using cyanogen bromide (CNBr), the variable C-terminal domain remains intact for TDP analysis (Figure 1). The C-terminal fragments of periostin isoforms range from 12.1 to 25.0 kDa in size, which is much more suitable for TDP analysis than intact periostin (Compton et al., 2011). These C-terminal fragments are unique for the respective isoforms allowing identification of individual periostin isoforms based on their detection in a TDP analysis. In addition to releasing the isoform specific C-terminal fragment, CNBr cleavage of periostin generates a fragment that is shared by all isoforms (_545_TSEE … SDIM_615_) termed the “shared fragment”. Here we present a TDP MS-based method for identifying periostin isoforms.

## 2 Methods and materials

### 2.1 Expression and purification of recombinant periostin

Recombinant periostin isoforms 1-10 were expressed and purified as previously described (Rusbjerg-Weberskov et al., 2023). Briefly, the recombinant periostin isoforms were expressed in a truncated form containing an N-terminal Twin-Strep-tag^®^, a TEV cleavage site, the FAS1-4 domain, and the C-terminal domain. The coding sequences were placed in-frame with the PelB signal peptide into the pET-22b (+) vector and expressed in One Shot™ BL21 (DE3) Chemically Competent *E. coli* (Thermo Fisher Scientific) in Terrific Broth with addition of 0.5 mM isopropyl β-D-1-thiogalactopyranoside (IPTG) at an OD_600_ of 1.0 to induce expression. Cells were pelleted by centrifugation and sonicated in the presence of c0mplete protease inhibitor cocktail. The cell lysate was filtered and recombinant periostin was purified on a StrepTactin®XT 4Flow^®^ 1 mL column (IBA Lifesciences).

### 2.2 CNBr cleavage of recombinant periostin

Purified recombinant periostin isoforms 1-10 were precipitated using trichloroacetic acid (TCA) to remove salts and chemically cleaved at their Met residues by CNBr. The purified isoforms (250 μg, 0.1–0.27 mg/mL) were added ice cold 80% TCA to a final concentration of 20% TCA, vortexed thoroughly, and incubated on ice for 90 min. Proteins were pelleted by centrifugation at 17.000 *g* for 30 min at 4°C. Supernatant was removed and the pellet was washed in 150 µL ethanol by vortexing and centrifugation. Ethanol was removed and the pellet was dried for 2 min at RT. The pellet was dissolved in 100 µL 70% trifluoroacetic acid (TFA) and added 100 µL CNBr cleavage solution (0.66 M CNBr, 70%, TFA, 13% acetonitrile). The samples were vortexed and incubated overnight at RT protected from light. CNBr solution was evaporated in a vacuum bell in the fume hood and samples were washed twice in 100 µL water and dried down in a speed-vac between washes. The washed samples were resolubilized in solvent A (0.1% formic acid, MS grade) and used directly for MS analysis. CNBr is a hazardous chemical and all recommended precautions were taken when handling CNBr. Waste containing CNBr was properly disposed of.

### 2.3 SDS-PAGE of recombinant periostin

The purified recombinant isoforms were analyzed by sodium dodecyl-sulfate polyacrylamide gel electrophoresis (SDS-PAGE). For each individually purified recombinant isoform, 5 µg was reduced by boiling 5 min in 1% SDS with 35 mM dithiotreitol and loaded on the gel. Acrylamide gels were cast in-house with a 5%–15% gradient and were stained with Coomassie following gel electrophoresis.

### 2.4 LC-MS and LC-MS/MS analyses

All liquid chromatography-mass spectrometry (LC-MS) and liquid chromatography tandem mass spectrometry (LC-MS/MS) experiments were performed on an Orbitrap Eclipse™ Tribrid™ Mass Spectrometer (Thermo Fisher Scientific) coupled to an Easy-nLC 1200 (Thermo FisherScientific) with a 6 cm trap column and a 15 cm analytical column packed in-house with ReproSil-Pur 300 C4, 3 µm (Dr. Maisch GmbH). All MS methods had the same global settings: Application mode set to intact protein, nanospray ionization source, spray voltage set to static, positive ion 1800 V, no sweep gas, ion transfer tube temperature set to 275°C, and standard pressure. The specific experimental settings are detailed below.

The LC method underwent minor optimization during the course of our work. For the screening of fragmentation methods, a 20 min gradient from 5% solvent B (80% acetonitrile, 0.1% formic acid) to 50% solvent B at 250 nL/min followed by 4 min at 95% solvent B was used. For all other experiments, a 20 min gradient from 25% solvent B to 88% solvent B at 250 nL/min followed by 4 min at 100% solvent B was used.

### 2.5 LC-MS analysis of individual recombinant isoforms

The CNBr cleaved recombinant isoforms were dissolved in solvent A to a concentration of 0.3–0.7 mg/mL. In each experiment, 1 µg was used. Full MS scans were obtained with the following parameters: Orbitrap resolution 120 K, quadrupole scan range 350–2000 m/z, RF lens 30%, AGC target of 1000%, max. injection time set to auto, 10 µscans, and data type set to profile. The aim of this experiment was to identify the most suitable precursor ions from each isoform to use in subsequent targeted MS/MS (tMS2) experiments, thus no MS/MS was performed in this experiment.

The acquired data was inspected in FreeStyle™ 1.8 SP2 (Thermo Fisher Scientific). The MS spectra were deconvoluted using FLASHDeconv software (Jeong et al., 2020) with the default parameters. Deconvoluted spectra were inspected in TOPPView and deviation from theoretical mass was calculated and reported along with other essential metrics in Table 1.

**TABLE 1:** The individual recombinant CNBr cleaved isoforms were successfully analyzed by high-resolution LC-MS. The table displays the observed monoisotopic mass from deconvolution of the respective MS spectra for each isoform. The theoretical monoisotopic mass and the deviation between observed and theoretical mass is shown. Multiple charge states were observed for each isoform.

Fragment	Observed mass, monoisotopic	Theoretical mass, monoisotopic	Mass deviation (ppm)	Observed charge states
Isoform 1 CTD	24994.83	24994.85	0.8	22-40
Isoform 2 CTD	18705.42	18705.30	6.4	13-31
Isoform 3 CTD	18938.52	18938.48	2.1	13-31
Isoform 4 CTD	15536.67	15536.66	0.6	9-26
Isoform 5 CTD	22107.22	22107.12	4.5	25-34
Isoform 6 CTD	15214.34	15214.29	3.3	11-25
Isoform 7 CTD	12045.66	12045.64	1.7	8-20
Isoform 8 CTD	21826.24	21826.20	1.8	18-35
Isoform 9 CTD	21693.11	21693.05	2.8	20-35
Isoform 10 CTD	18524.44	18524.41	1.6	14-31

### 2.6 Targeted MS/MS fragmentation of individual recombinant isoforms

All CNBr cleaved recombinant isoforms were subsequently analyzed by targeted MS/MS (tMS2). Different fragmentation methods (collision-induced dissociation (CID), higher-energy collisional dissociation (HCD), electron transfer dissociation (ETD), and electron-transfer/higher-energy collisional dissociation (EThcD)) were tested for each isoform. Purified CNBr-cleaved isoforms were injected separately, and the fragmentation method and targeted precursor ion were optimized for each isoform. For each experiment 1 µg of protein was injected with the following MS parameters: Quadrupole isolation with 3 m/z isolation window, orbitrap resolution 120 K, RF lens 60%, normalized AGC target of 1000%, max. injection time set to auto, 10 µscans, and data type set to profile. One precursor ion per isoform was selected for tMS2 experiments (precursor m/z and charge state information is shown in Supplementary Table S1). Fragmentation optimization was tested initially on isoforms 3 and 4, which were analyzed individually. MS parameters were as follows: CID with 30% for 10 ms, HCD with normalized energy at 40%, ETD with 10 ms reaction time, and EThcD with 20% HCD/20 ms ETD reaction time or 35% HCD/35 ms ETD reaction time. Data were processed using FreeStyle™ 1.8 SP2 (Thermo Fisher Scientific) and ProSight Lite (Fellers et al., 2015) as described below.

Analysis of this set of experiments by ProSight Lite showed that fragmentation using ETD10 (ETD with 10 ms reaction time) was the most efficient for isoform fragmentation. For the next set of experiments, ETD with reaction times of 5, 10, 15, or 20 ms with 2*10^5^ ETD reagent target and max. ETD reagent injection time of 200 ms was then used to fragment isoforms 3 and 10 separately. For isoforms 3 and 10, the fragmentation was most complete when ETD5 was used. All ten isoforms were individually analyzed with tMS2 ETD5 to confirm its efficiency across all isoforms. Based on these results, ETD5 was chosen for all subsequent analyses.

### 2.7 Processing of LC-MS/MS data in FreeStyle™ and ProSight lite

Raw MS files were inspected in FreeStyle™ 1.8 SP2 (Thermo Fisher Scientific). Averaged MS/MS spectra based on the observed retention time of the individual isoforms from the LC-MS experiment were deconvoluted using the built-in Xtract algorithm. The mass lists were exported and pasted into the ProSight Lite (Fellers et al., 2015) application. The sequence of the C-terminal domain (_616_TTNG … ) released after CNBr cleavage for the respective isoform, the observed mass from the LC-MS experiment, and the relevant fragmentation method were entered in the ProSight Lite analysis. Fragment mass tolerance was set to 10 ppm. For the shared fragment (_545_TSEE … SDIM_615_), the C-terminal methionine residue was modified to a homoserine lactone (−48.00337 Da) occurring from CNBr cleavage.

### 2.8 LC-MS/MS analysis of a mix of all ten recombinant isoforms

Equal amounts of all ten CNBr cleaved recombinant isoform samples were pooled for subsequent LC-MS/MS analysis using the optimal fragmentation method, ETD5. Two different approaches were tested: Parallel reaction monitoring (PRM) and multiplexing. A total of 1 µg was loaded of the pool, thus each isoform being approximatively 10 times diluted compared to the analyses of individual isoforms. The targeted precursors and isolation window were adjusted in both methods to avoid co-isolation of precursors. The targeted precursor ions for the PRM and multiplexing experiments are listed in Supplementary Table S1. The specific parameters for the different methods are described below.

PRM: MS scan with orbitrap resolution 120 K, quadrupole scan range 350–2000 m/z, RF lens 60%, AGC target of 1000%, max. injection time set to 50 ms, 1 µscan, and data type set to profile. Parameters for single precursor MS/MS scans were quadrupole isolation with 1.2 m/z isolation window, orbitrap resolution 120 K, RF lens 60%, AGC target of 1000%, max. injection time set to 150 ms, 1 µscan, and data type set to profile. Each cycle contains a MS master scan followed by MS/MS of the inclusion list of isoforms.

Multiplexing: MS scan with orbitrap resolution 120 K, quadrupole scan range 350–2000 m/z, RF lens 60%, AGC target of 1000%, max. injection time set to 50 ms, 2 µscans, and data type set to profile. Parameters for multiplexing MS/MS scans were quadrupole isolation with 1 m/z isolation window, orbitrap resolution 120 K, RF lens 60%, AGC target of 1000%, max. injection time set to 150 ms, 2 µscans, and data type set to profile. The three precursors belonging to the same isoform were grouped and isolated for combined fragmentation. Each cycle contains a MS master scan followed by MS/MS of the list of isoform groups.

## 3 Results

### 3.1 Recombinant periostin isoforms used as model system

**FIGURE 2:**
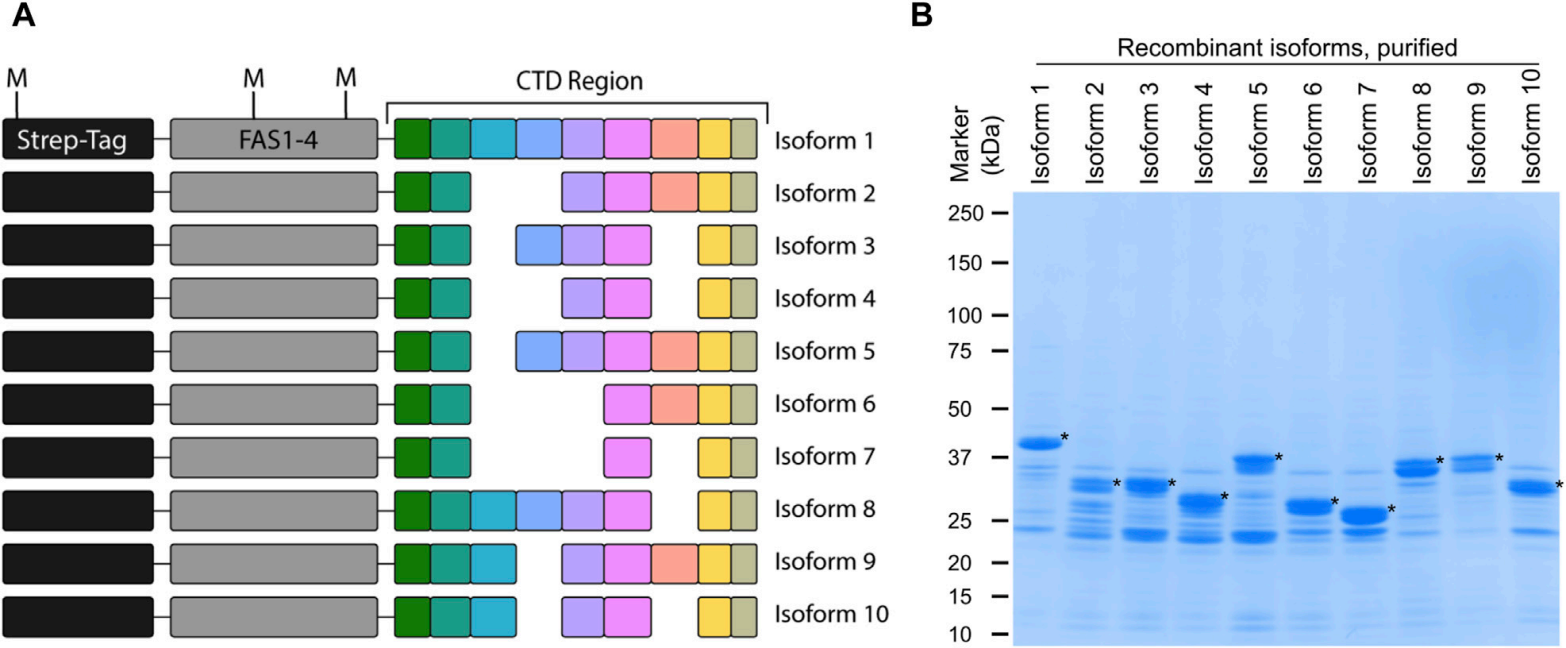
Recombinant periostin is used as a model system to develop a top-down MS-based assay for isoform identification. **(A)** Schematic overview of the recombinant periostin containing an N-terminal Twin-Strep-tag^®^, the FAS1-4 domain, and the C-terminal domain. All ten periostin isoforms were expressed and purified with this design as illustrated. The position of the methionine residues (M) is indicated. **(B)** SDS-PAGE analysis of each of the purified recombinant periostin constructs. The respective recombinant isoforms are marked with an asterisk (*). Recombinant periostin isoforms are successfully produced and purified to a satisfactory level of purity for the application of TDP method development.

Identification of periostin isoforms produced by alternative splicing at the protein level is a challenge due to their high degree of similarity. By employing top-down MS, virtually any proteoform can be distinguished. Here we introduce a novel top-down MS-based approach to identify periostin splice isoforms. To develop this assay, a model system based on recombinant periostin isoforms was established. Recombinant periostin containing an N-terminal tag, the last FAS1 domain, and the variable C-terminal domain (Figure 2A) was expressed in *E. coli* and enriched by affinity chromatography. The enriched samples were analyzed by SDS-PAGE, where the isoforms can be identified at the expected molecular weight and appear as the most abundant species in the sample (Figure 2B). The presence of contaminants does not pose a problem for the use of the recombinant proteins as a model for development of an isoform specific TDP method.

**FIGURE 3:**
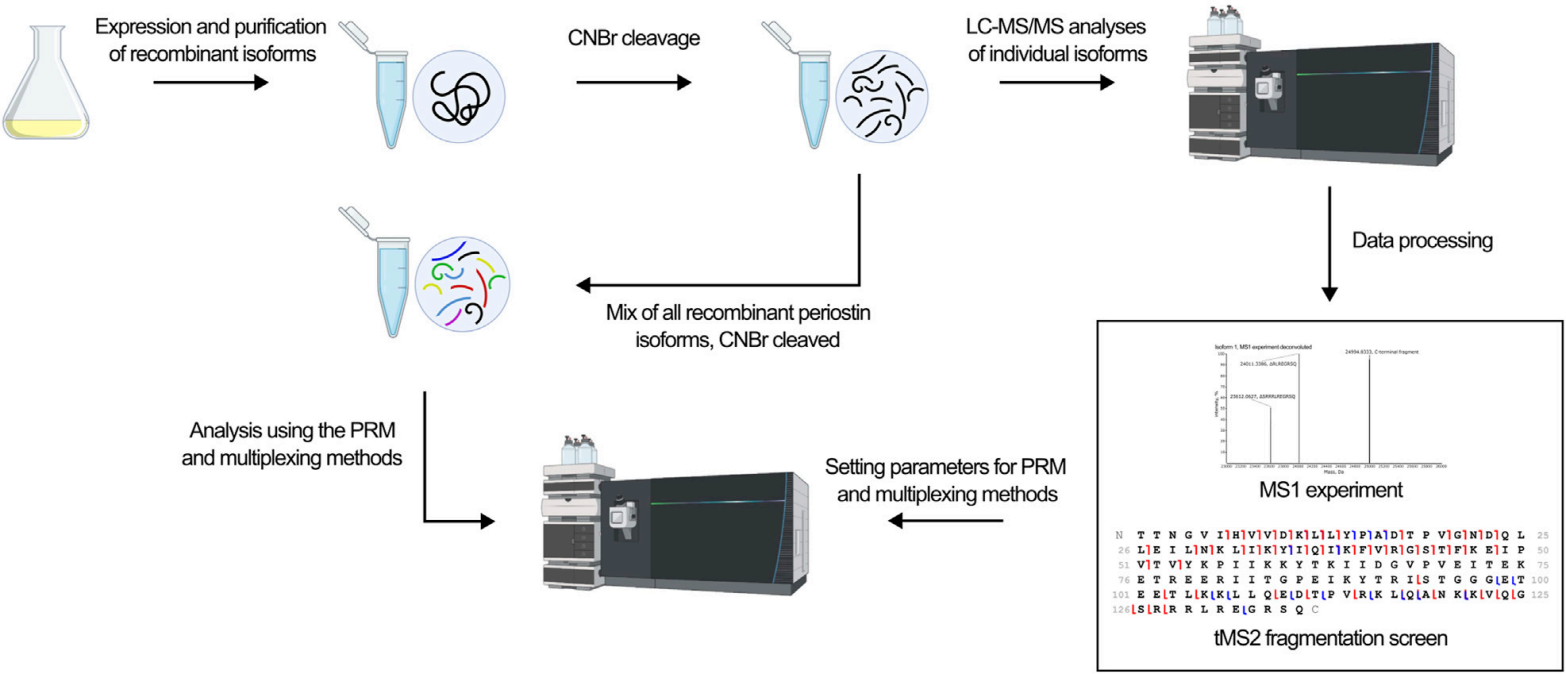
The workflow in the development of an isoform specific top-down assay is outlined. Recombinant isoforms were expressed individually and purified. Following CNBr cleavage, the isoforms were analyzed individually in first a MS1 experiment to identify the precursor ions to target and second a tMS2 fragmentation screen to identify the optimal fragmentation method. Based on these xperiments, the optimal fragmentation method was found and used in the development of a PRM and multiplexing method. Ultimately, the ability of the methods to identify all isoforms from a mixture of all ten isoforms was tested.

The development of a top-down MS-based assay for identification of periostin isoforms follows the workflow illustrated in Figure 3. The assay employs targeted LC-MS/MS of the C-terminal fragment released by CNBr cleavage of periostin. First, an MS1 experiment of the individual CNBr cleaved recombinant isoforms provides an overview of the observed precursor ions for each isoform and their relative intensity and charge states. Based on the MS1 experiment, a target precursor for each isoform is selected for LC-MS/MS fragmentation screening. The isoforms were analyzed individually with different fragmentation methods to find the optimal fragmentation method for the assay. Two different approaches were then tested for their ability to confidently identify all isoforms from a mixture of the CNBr cleaved recombinant isoforms.

### 3.2 High-resolution MS1 identifies multiple charge states for each isoform

**FIGURE 4:**
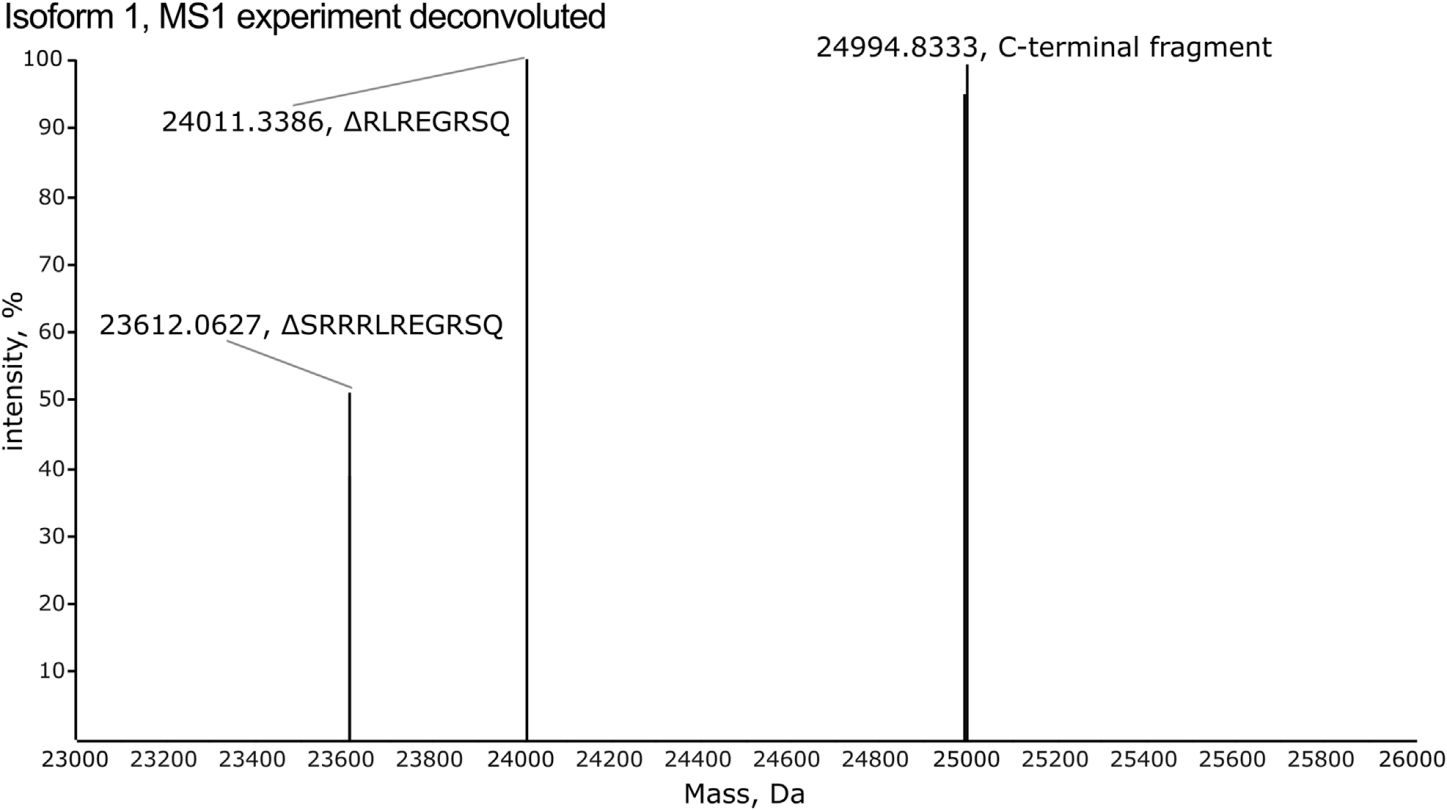
The deconvoluted spectrum from the MS1 experiment of isoform 1 reveals detection of the C-terminal fragment released by CNBr cleavage. Two additional fragments are observed, which correspond to C-terminally truncated versions of the C-terminal fragment. These lack the eight (RLREGRSQ) and eleven (SRRRLREGRSQ) most C-terminal residues, respectively.

All isoforms were analyzed individually by high-resolution MS1 (120 K orbitrap resolution) in order to compare the intensity of the individual charge states for each isoform and to identify the observed m/z values to target in the subsequent PRM and multiplexing LC-MS/MS analyses. To inspect the fragments generated by CNBr cleavage, MS1 spectra were deconvoluted. The deconvoluted spectrum of isoform 1 displays an intense peak corresponding to the theoretical monoisotopic mass of the C-terminal fragment produced by CNBr cleavage with a deviation of 0.8 ppm (Figure 4). The C-terminal fragment was also detected with high accuracy of 0.6–6.4 ppm for the other nine isoforms (Supplementary Figures S1–S3). The observed and theoretical monoisotopic masses, the mass deviation, and the observed charge state of each isoform are listed in Table 1. Additionally, two other fragments are observed in the deconvoluted spectrum of isoform 1 that correspond to the C-terminal fragment, which have been C-terminally truncated (Figure 4). The same truncations are observed for all ten recombinant isoforms except for isoform 5 (Supplementary Figures S1–S3) despite the inclusion of protease inhibitors in the preparation of the recombinant proteins. The intact C-terminal fragment is the most intense in most cases and the samples can reliably be used for the development of the TDP method in scope. In addition to the C-terminal fragment, the shared fragment is also observed in all of the MS1 experiments with its C-terminal methionine residue converted to homoserine lactone (Supplementary Table S2).

### 3.3 Electron transfer dissociation produces high sequence coverage for all isoforms

**FIGURE 5:**
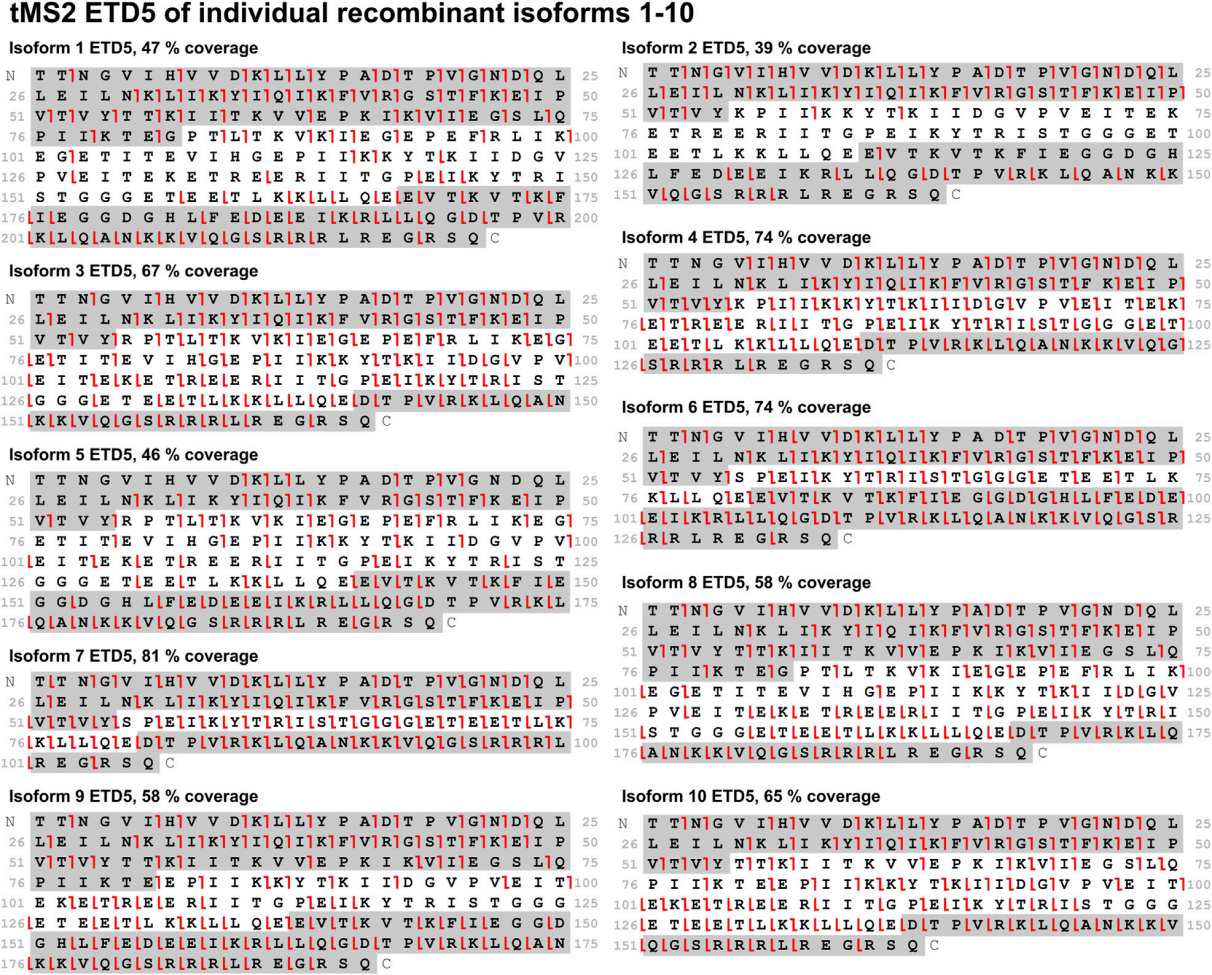
All ten isoforms were subjected to tMS2 fragmentation screening of ETD5. Sequence coverage is displayed above each fragmentation map. Fragments from the sequence highlighted in grey (c-ions in the C-terminal grey highlight, z-ions in the N-terminal grey highlight) are unique to the respective isoform. Red lines indicate c-/z-ions. Fragment tolerance is 10 ppm. All isoforms display comprehensive sequence coverage and distribution of fragments across the sequence.

With the knowledge on the most intense charge states and their observed m/z values for each isoform, a tMS2 fragmentation screening was performed. The initial screening of CID, HCD, EThcD, and ETD using isoforms 3 and 4 showed that ETD is the most suitable fragmentation method for this assay. ET35hcD35 gave high sequence coverage but EThcD generally favored fragmentation of the shared N- and C-terminal part (Supplementary Figure S4). CID and HCD also favored fragmentation of the termini but also produced fragments throughout the entire sequence (Supplementary Figure S4). The sequence coverage from ETD10 stood out among the techniques and produced fragments across the entire sequence (Supplementary Figure S4). Expanding the screening to different reaction times of ETD to 5, 10, 15, and 20 ms using isoforms 3 and 10 identified an ETD reaction time of 5 and 10 ms as the most efficient, while prolonged reaction times led to over fragmentation and loss of identification of the internal fragments (Supplementary Figure S5). ETD5 and ETD10 performed similarly well with a small advantage to ETD5 in terms of sequence coverage (Supplementary Figure S2), and therefore ETD5 was chosen as the fragmentation method for the isoform specific TDP assay. Analyzing all CNBr cleaved recombinant isoforms individually with tMS2 using ETD5 fragmentation confirmed its efficiency for this assay (Figure 5). A high sequence coverage ranging from 39% to 81% with fragments distributed throughout the sequence is observed with ETD5 (Figure 5).

### 3.4 Two approaches to identify isoforms from a mixture

**FIGURE 6:**
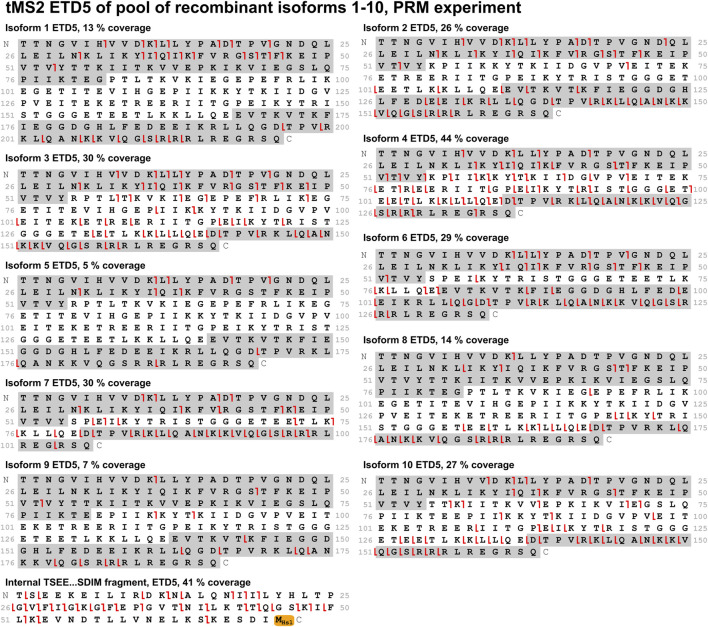
A mix of all ten isoforms was analyzed with the PRM method using ETD5 fragmentation. Sequence coverage is displayed above each fragmentation map. Fragments from the sequence highlighted in grey (c-ions in the C-terminal grey highlight, z-ions in the N-terminal grey highlight) are unique to the respective isoform. The shared fragment with its C-terminal methionine residue modified to a homoserine lactone (−48.00337 Da) is also shown. Red lines indicate c-/z-ions. Fragment tolerance is 10 ppm. All isoforms were successfully identified in this experiment.

**FIGURE 7:**
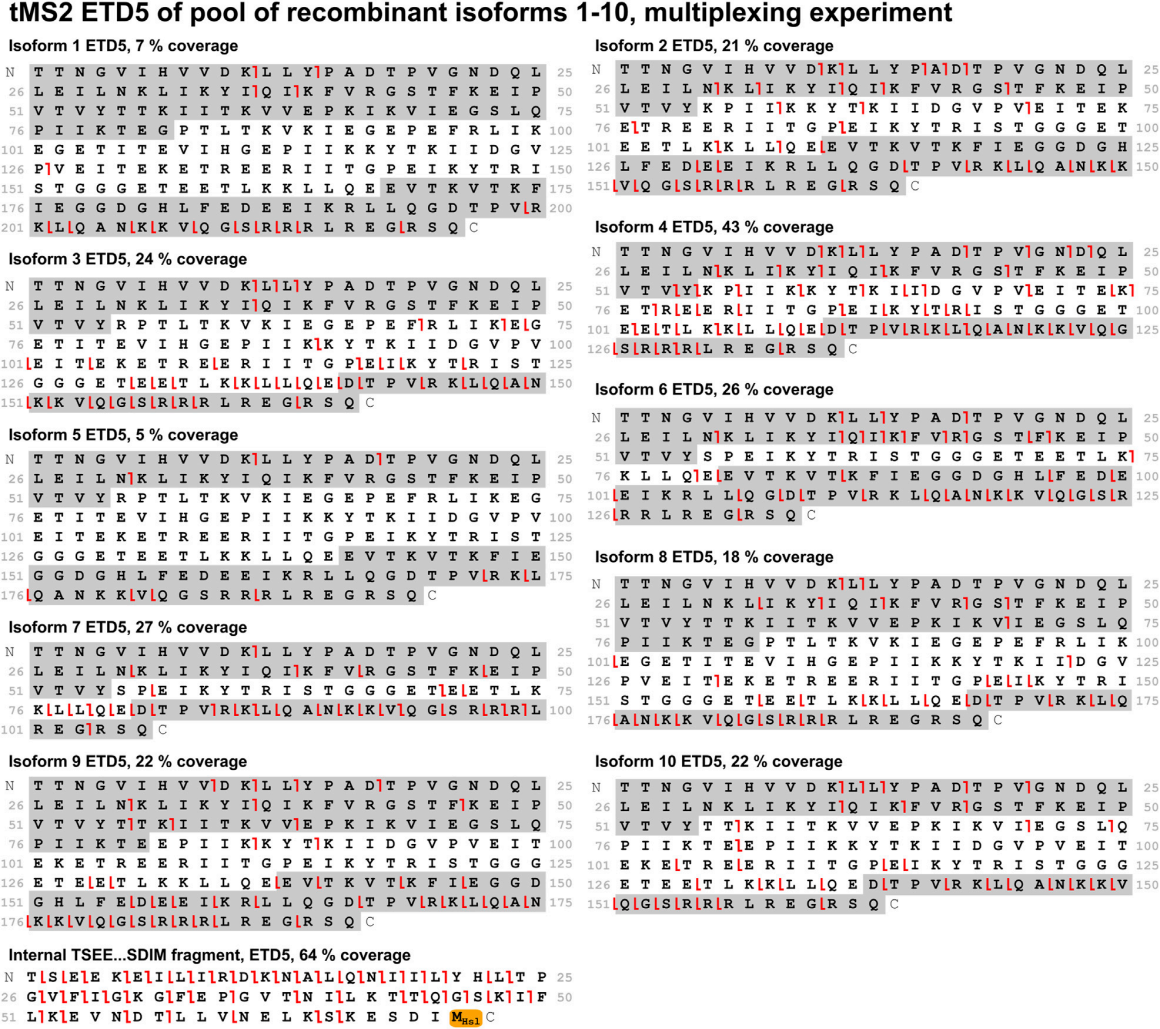
A mix of all ten isoforms was analyzed with the multiplexing method using ETD5 fragmentation. Sequence coverage is displayed above each fragmentation map. Fragments from the sequence highlighted in grey (c-ions in the C-terminal grey highlight, z-ions in the N-terminal grey highlight) are unique to the respective isoform. The shared fragment with its C-terminal methionine residue modified to a homoserine lactone (−48.00337 Da) is also shown. Red lines indicate c-/z-ions. Fragment tolerance is 10 ppm. All isoforms were successfully identified in this experiment.

With the optimal fragmentation method in place, the focus was turned to develop a method that could identify all ten isoforms from a mixture of these. Two different approaches were compared: A PRM method and a multiplexing method. In the PRM method, the mass spectrometer sequentially isolates the individual isoforms for MS/MS throughout the entire experiment in a defined cycle, which also makes this method suitable for relative quantification analysis. The multiplexing method is analogous to the PRM method. It differs by isolating three precursors of each isoform for MS/MS, that is, three different charge states of the same isoform. This improves sensitivity and comes with the payoff of a longer cycle time making it less suitable for quantification. Comparing these methods when analyzing a mix of all ten isoforms, the PRM method performed better as it gave higher sequence coverage for most isoforms. Both methods provided high sequence coverage of the shared fragment with 41% and 64% for the PRM and multiplexing method, respectively (Figures 6, 7). In comparison to the tMS2 analysis of the individual isoforms (Figure 5), the decrease in sequence coverage observed in the PRM and multiplexing methods (Figures 6, 7) is owed to loading one-10th of each isoform and decreased instrument time for each isoform. Overall, both methods were able to successfully identify all ten isoforms from a mixture.

### 3.5 Discussion

We began this study with the aim of developing a top-down MS method that could uniquely identify all ten periostin isoforms at the protein level in a complex sample. Using the recombinant model, the target precursor ions for each isoform were characterized, a fragmentation screening was performed, and the efficiency of different approaches were tested in order to develop a PRM method for the identification of periostin isoforms.

The recombinant model system designed for this study produces the exact same fragments of interest following CNBr cleavage that is also produced by CNBr cleavage of endogenous periostin from any human tissue or cell culture sample. Thus, the presented top-down MS workflow could be transferable for use on more complex samples containing periostin. In addition to the C-terminal fragment produced by CNBr cleavage of periostin, the shared fragment also generated from CNBr cleavage of endogenous periostin can be used as internal reference peptide for relative quantification of total periostin in application of the presented method. The uniqueness of this shared fragment was confirmed in a BLAST analysis. An additional fragment containing the N-terminal Twin-Strep-tag^®^ is produced by CNBr cleavage of each of the recombinant periostin isoforms. This fragment has no relevance in the application of the method and was therefore not included in our analyses. It should be noted that the mechanism of CNBr cleavage modifies the C-terminal methionine residue to become a homoserine or homoserine lactone (Gross and Witkop, 1962). The shared fragment in which the methionine residue is converted to a homoserine lactone was much more intense than the homoserine counterpart.

A challenge in the design of the targeted assays has been to identify the optimal precursor to target for each isoform. The many observed charge states for each isoform (Table 1) create a complex matrix of precursors in the range of 700–950 m/z in which the most intense charge states are observed (Supplementary Table S3). This poses a risk of co-isolating another isoform in the targeted assay. Due to the shared N- and C-termini, co-isolation of precursors with similar m/z value from different isoforms could potentially lead to false negative identifications at the MS/MS level. This issue of co-isolation is even more extensive in the multiplexing approach where multiple m/z are targeted per experiment and the isolation window had to be narrowed down to 1 m/z. This challenge accounts for the difference in precursors targeted in the fragmentation screening experiment, where the isoforms were analyzed individually without risk of co-isolation, and the PRM and multiplexing assay (Supplementary Table S1). The closest m/z value of another isoform is listed for each targeted precursor in the PRM assay in Supplementary Table S4. The isolation window is set to 1.2 and 1 m/z for the PRM and multiplexing method, respectively, and the smallest difference between a targeted precursor and any precursor from any isoform is >1.5 and >1 m/z for the PRM and multiplexing method, respectively (Supplementary Tables S3, S4).

In application of the assay presented in this work it is not necessary to obtain high sequence coverage and unique fragment ions to achieve confident identification of an isoform. Generally, fragment ions from the shared N- and C-termini have been the most intense. Since experimentally observed precursors are shown to be selectively isolated in this targeted assay without any risk of co-isolation, the observation of fragment ions from the shared termini is sufficient for isoform identification. Isoforms can also be isolated according to their retention time by using a narrow selection window in the MS method.

The PRM and the multiplexing methods were comparable in terms of sequence coverage with a slight advantage to the PRM method. As described above, the multiplexing method required selection of less intense charge states for targeting to avoid co-isolation. Had it been possible to target the top three most intense charge states for each isoform, the multiplexing method had likely outperformed the PRM method. This is seen for the shared fragment where the top three charge states could be targeted to give a 64% sequence coverage that is substantially higher than the 41% obtained with the PRM method. An advantage for the PRM method is its faster cycle time, which gives more scans that enable more points to enable relative quantification. Thus, we present the PRM method as a highly sensitive tool to identify and quantify periostin isoforms in human samples after CNBr cleavage.

Discrepancy between mRNA and protein levels of periostin have been reported (Morra et al., 2012) underlining the importance of characterizing periostin splice isoforms at the protein level. Additionally, RT-qPCR and other mRNA-based methods are limited to analyze cellular tissues in which the periostin expressing cells are contained. The analysis of periostin in acellular fluids like plasma, saliva, or synovial fluid requires methods that operate at the protein level. Periostin is used as biomarker for multiple diseases (Ben et al., 2009; Izuhara et al., 2016; Sung et al., 2017; Azharuddin et al., 2019; Massy et al., 2021), and it is part of the pathogenesis of periodontitis (Yamada et al., 2014) and osteoarthritis (Honsawek et al., 2015; Attur et al., 2021; Duan et al., 2021). Insight into the periostin isoform profile in these diseases is essential to understand the role of periostin, but currently no assays exist that can exhaustively characterize the periostin isoforms in the plasma, saliva, and synovial fluid samples relevant to these diseases. The top-down method presented here can provide a detailed characterization of the periostin splice profile in a human sample of interest at the protein level. Thus, the assay holds the potential to improve the foundation for the understanding of the periostin isoforms. The disordered C-terminal domain of periostin has an extensive interactome and has previously been proposed to act as a scaffold (Rusbjerg-Weberskov et al., 2023). The interactome of the modular C-terminal scaffold is hypothesized to be regulated by alternative splicing that ultimately shapes periostin function. We hypothesize that the key to fully understand periostin in health and disease lies in the variation of the C-terminal domain. Other studies will address the functional differences of the isoforms while this work presents a powerful tool for precise characterization of splice profiles.

In addition to the direct use of the presented assay for investigating periostin isoforms, our work demonstrates that incorporating a fragmentation step in combination with TDP can be used in the mapping of splice isoforms. TDP analysis of splice isoforms with high molecular weight or proteoforms of large proteins is challenging and the crucial information is lost upon cleavage into peptides for a BUP analysis. We show that a fragmentation step enables TDP analysis of a protein that was previously unsuitable for high-resolution TDP LC-MS/MS analysis. Using chemical cleavage reagents or proteases that cleave proteins at less common amino acid residues or rare motifs can generate larger fragments encompassing the site of variation, making them suitable for TDP analysis. In this study, we used CNBr, but other chemical cleavage reagents such as BNPS-skatole (which cleaves at tryptophan residues) and hydroxylamine (which cleaves at asparagine-glycine motifs) would have produced nearly the same periostin fragment. Alternatives include 2-nitro-5-thiocyanobenzoic acid (NTCB) cleaving at the site of cysteine residues or proteases cleaving at rare motifs. The choice of cleavage depends on the protein of interest to generate small fragments containing the variation of interest for TDP analysis.

As presented here, the assay is ready for implementation on any type of human sample. Depending on the sample/tissue type, the CNBr cleavage may produce highly complex samples that may pose a challenge for TDP analysis, even with the PRM approach. To address this, we are currently investigating the use of either a pre-cleavage enrichment step of periostin, e.g., by immunopurification or a post-cleavage enrichment step of the C-terminal region of periostin released by CNBr cleavage. The C-terminal domain of all isoforms contains an arginine-rich motif, RRRLREGRS, in the very terminal and is present in all isoforms (Figure 1). Thus, enrichment of the C-terminal regions after CNBr cleavage could rely on the heparin-binding ability inferred by this motif (Rusbjerg-Weberskov et al., 2023). The advantage of exploiting this common heparin-binding site is the hypothesized unbiased enrichment of all isoforms, which is essential for its compatibility with the assay. Similarly, an immunopurification step must be performed with antibodies recognizing regions shared by all isoforms, e.g., the CROPT and FAS1 domains, to avoid bias of enrichment of certain isoforms. However, the sequence variation of the C-terminal domains may influence heparin-binding strength, and they might shield epitopes and influence immunopurification. With the enrichment protocol in place we will be able to demonstrate the applicability of the presented method on an array of human samples.

## 4 Conclusion

Based on a recombinant model system, a top-down MS PRM method has successfully been developed. The initial MS1 experiment verified that the predicted C-terminal fragments of all periostin isoforms could be produced from CNBr cleavage of recombinant periostin. From the fragmentation screening, ETD was determined to be the method of choice to obtain high sequence coverage with a proper distribution of fragments across the sequence. A PRM method targeting a single precursor for each isoform and a multiplexing method targeting three precursors for each isoform were developed. The PRM method performed slightly better and has the advantage of an additional quantitative capacity. Thus, we present a top-down MS PRM method that has the potential to be applied to any type of human sample to uniquely identify and quantify the periostin splice isoforms in the sample. Application of this top-down MS assay can stand alone, complement RT-PCR-based analyses, or make it possible to characterize the periostin splice profile of extracellular samples like plasma, saliva, and synovial fluid devoid of transcriptional information.

## Data Availability

The datasets presented in this study can be found in online repositories. The names of the repository/repositories and accession number(s) can be found below: https://www.ebi.ac.uk/pride/archive/, PXD045560.

## Abbreviations

BUP, bottom-up proteomics; CID, collision-induced dissociation; CNBr, cyanogen bromide; ETD, electron transfer dissociation; EThcD, electron-transfer/higher-energy collisional dissociation; HCD, higher-energy collisional dissociation; LC-MS, liquid chromatography-mass spectrometry; LC-MS/MS, liquid chromatography tandem mass spectrometry; MS, mass spectrometry; PRM, parallel reaction monitoring; RT, room temperature; SDS-PAGE, sodium dodecyl-sulfate polyacrylamide gel electrophoresis; TCA, Trichloroacetic acid; TFA, trifluoroacetic acid; tMS2, targeted tandem mass spectrometry.
