# Supplementary material for: Development of a top-down MS assay for specific identification of human periostin isoforms
Source: Front Mol Biosci. 2024 Jun 19;11:1399225. doi: 10.3389/fmolb.2024.1399225 (PMC11220192; doi:10.3389/fmolb.2024.1399225)
Supplement: Supplementary file 1 [file DataSheet1.docx]

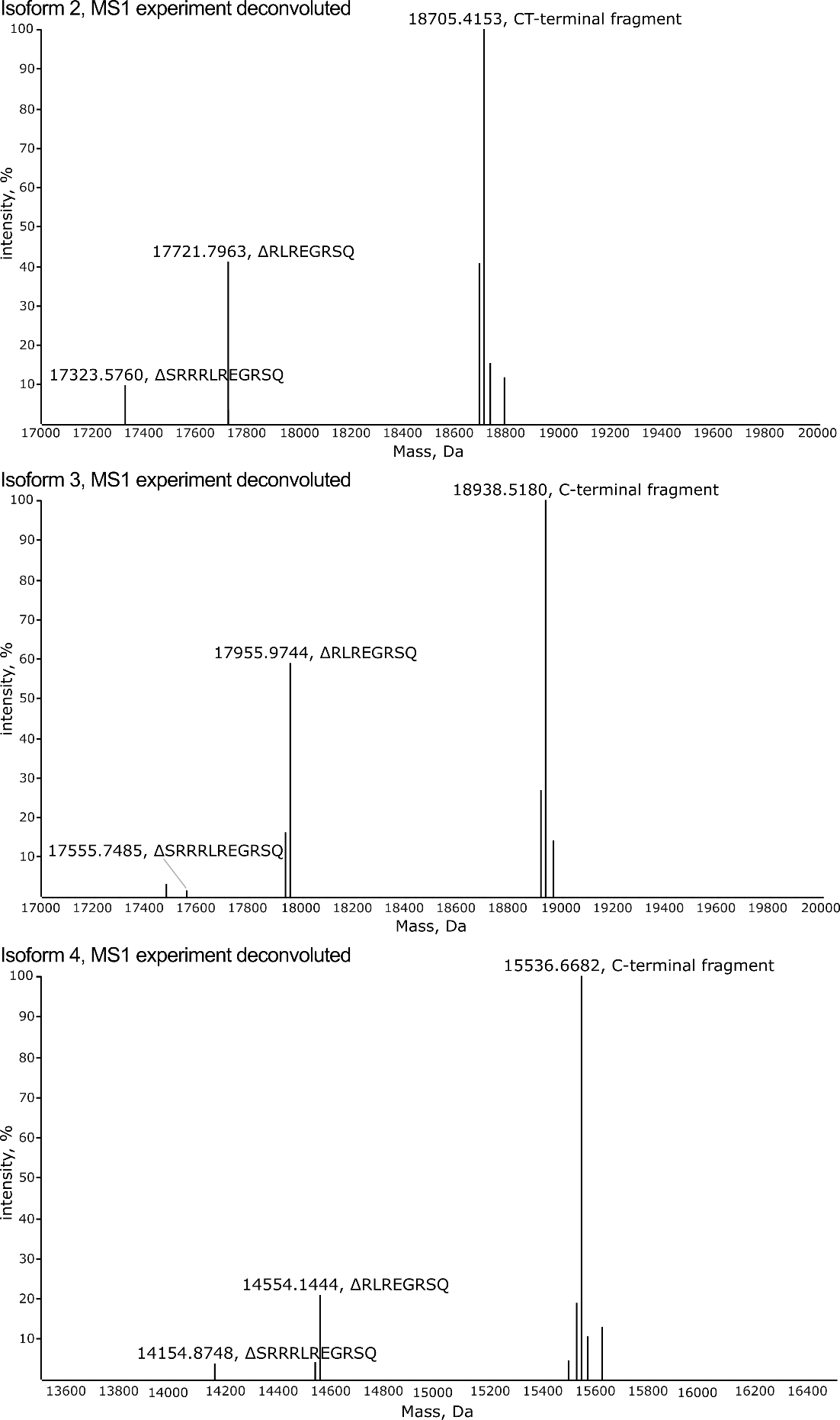


**Figure S1.** The deconvoluted spectra from the MS1 experiment of isoform 2-4 reveal detection of the C-terminal fragment releases by CNBr cleavage. Two additional fragments are observed, which correspond to C-terminally truncated versions of the C-terminal fragment. These lack the eight (RLREGRSQ) and eleven (SRRRLREGRSQ) most C-terminal residues, respectively.

**
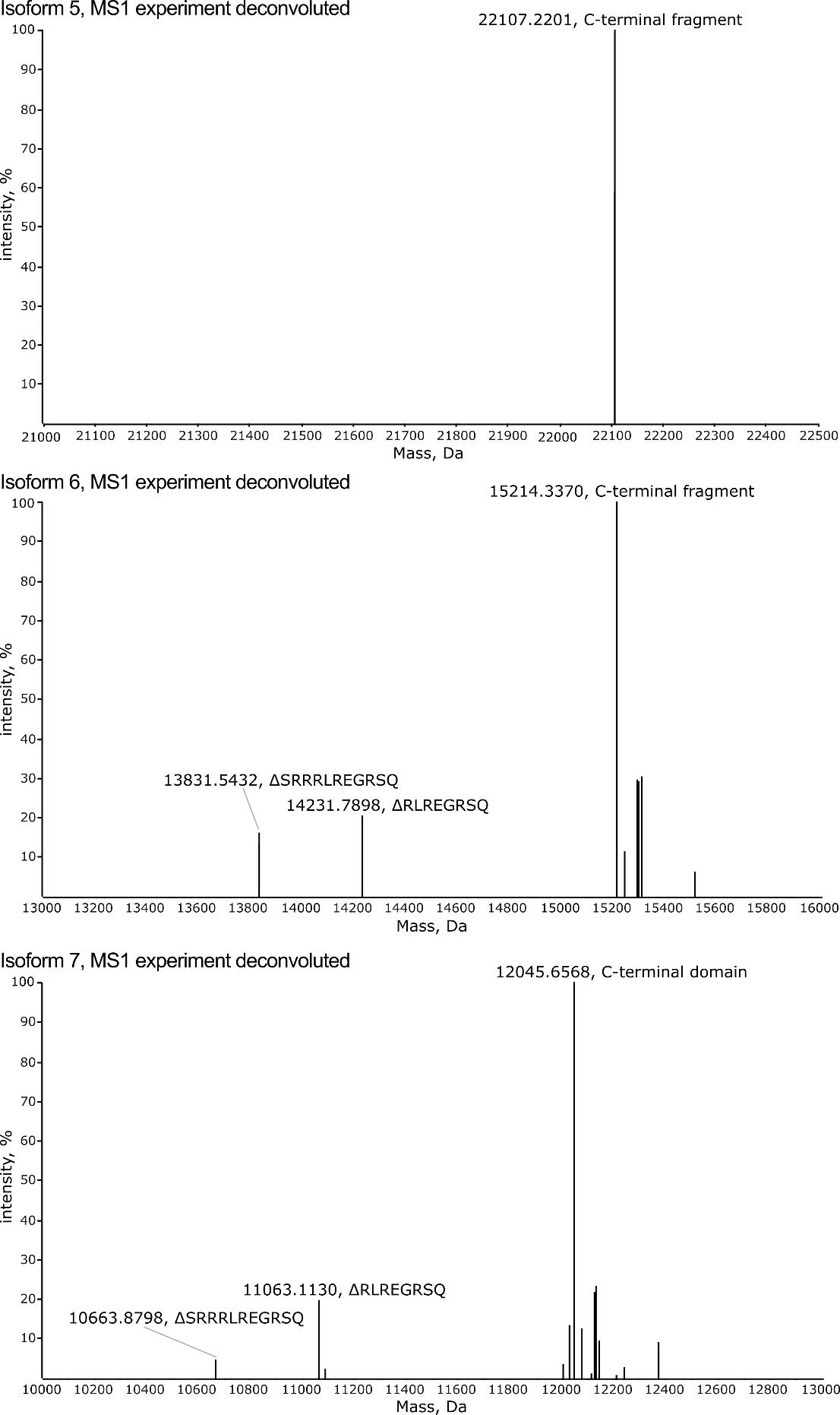
**

**Figure S2.** The deconvoluted spectra from the MS1 experiment of isoform 5-7 reveal detection of the C-terminal fragment releases by CNBr cleavage. Two additional fragments are observed (isoform 6 and 7), which correspond to C-terminally truncated versions of the C-terminal fragment. These lack the eight (RLREGRSQ) and eleven (SRRRLREGRSQ) most C-terminal residues, respectively.

**
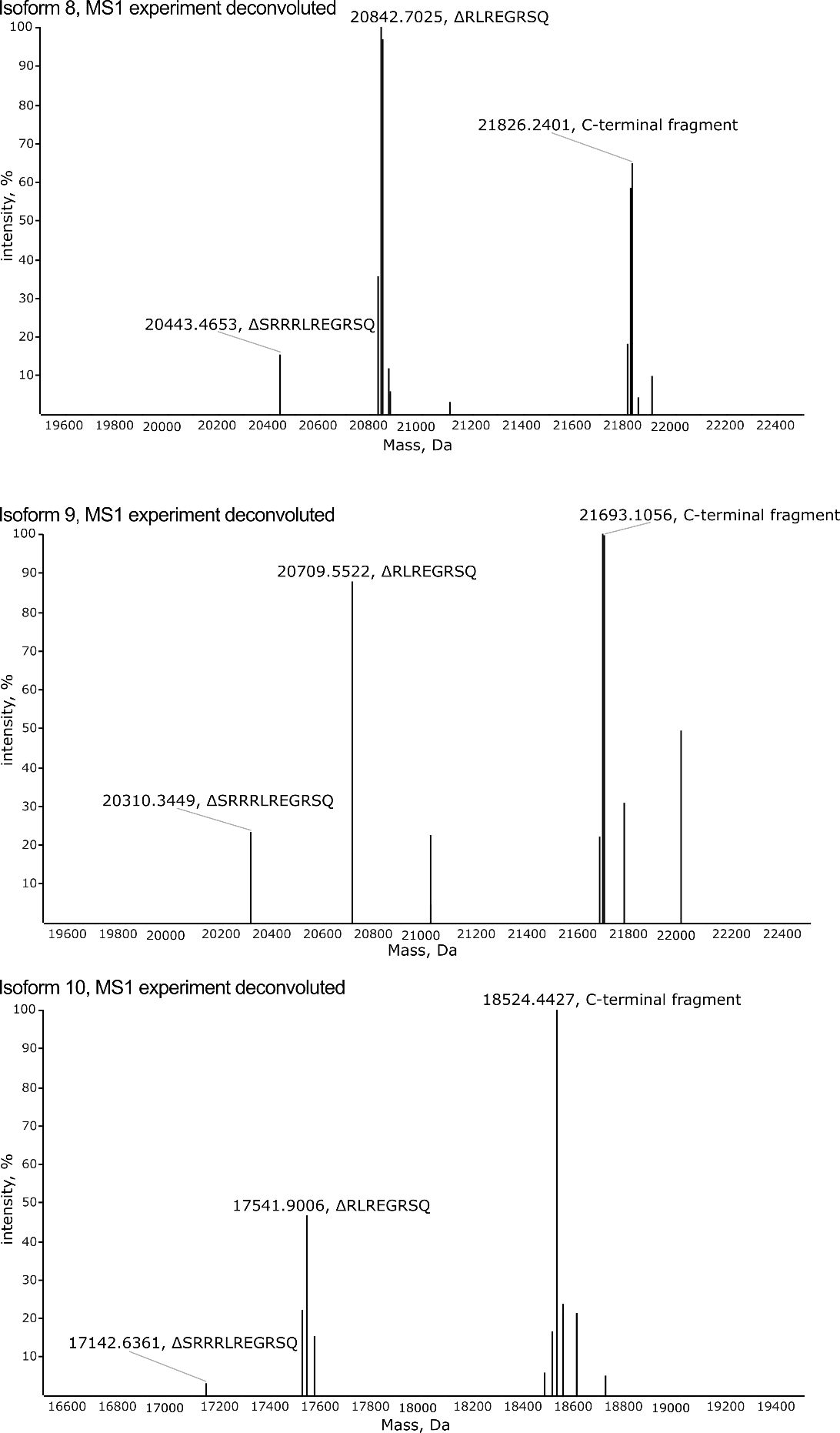
**

**Figure S3.** The deconvoluted spectra from the MS1 experiment of isoform 8-10 reveal detection of the C-terminal fragment releases by CNBr cleavage. Two additional fragments are observed, which correspond to C-terminally truncated versions of the C-terminal fragment. These lack the eight (RLREGRSQ) and eleven (SRRRLREGRSQ) most C-terminal residues, respectively.


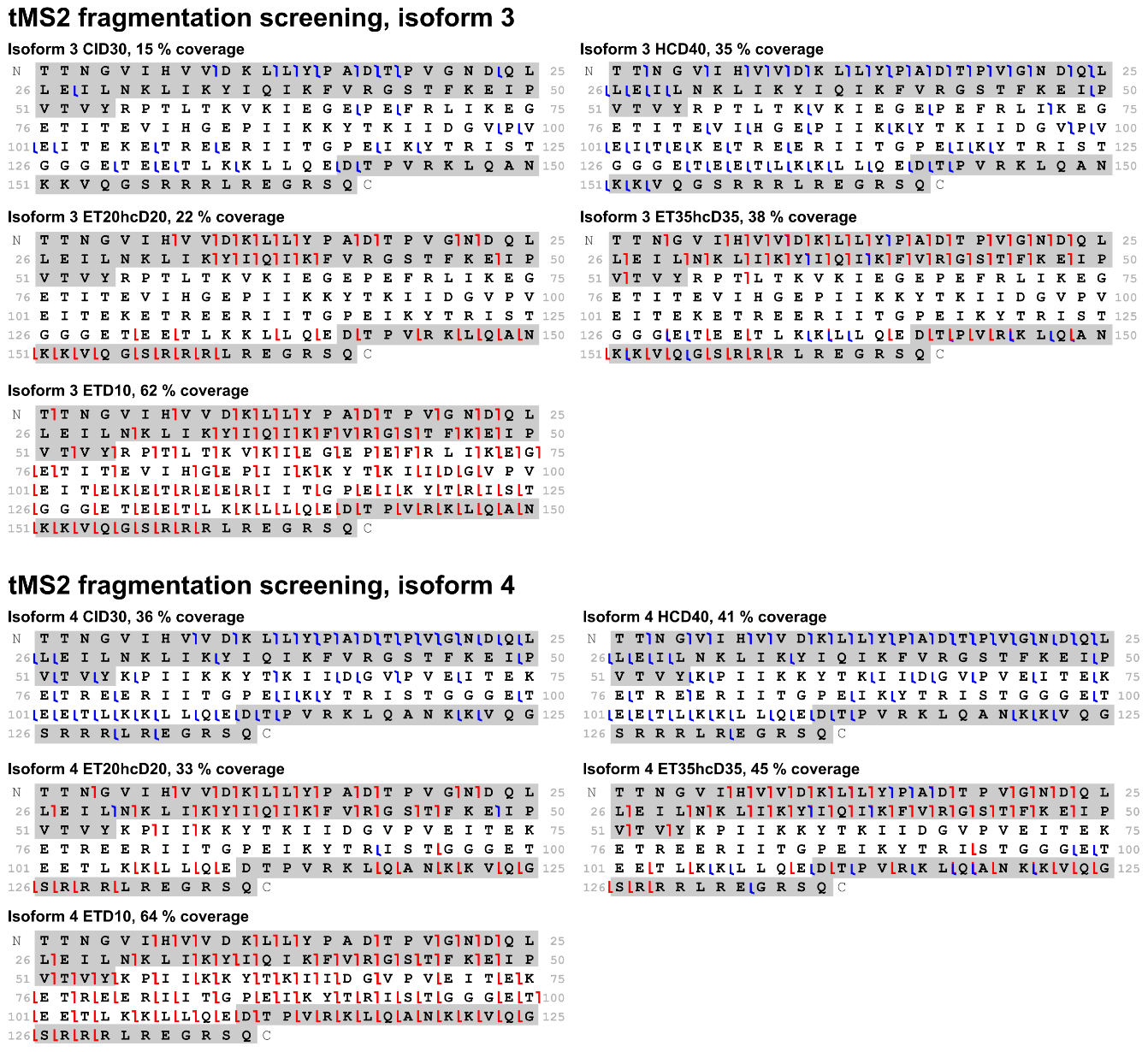


**Figure S4.** Isoforms 3 and 4 were subjected to tMS2 fragmentation screening of CID, HCD, EThcD, and ETD. Fragmentation technique and sequence coverage is displayed above each fragmentation map. Fragments from the sequence highlighted in grey (b/c-ions in the C-terminal grey highlight, y/z-ions in the N-terminal grey highlight) are unique to the respective isoform. Blue and red lines indicate b-/y-ions and c-/z-ions, respectively. Fragment tolerance is 10 ppm. For both isoforms, ETD10 outperforms the other fragmentation techniques in terms of sequence coverage and distribution of fragments across the sequence.

**
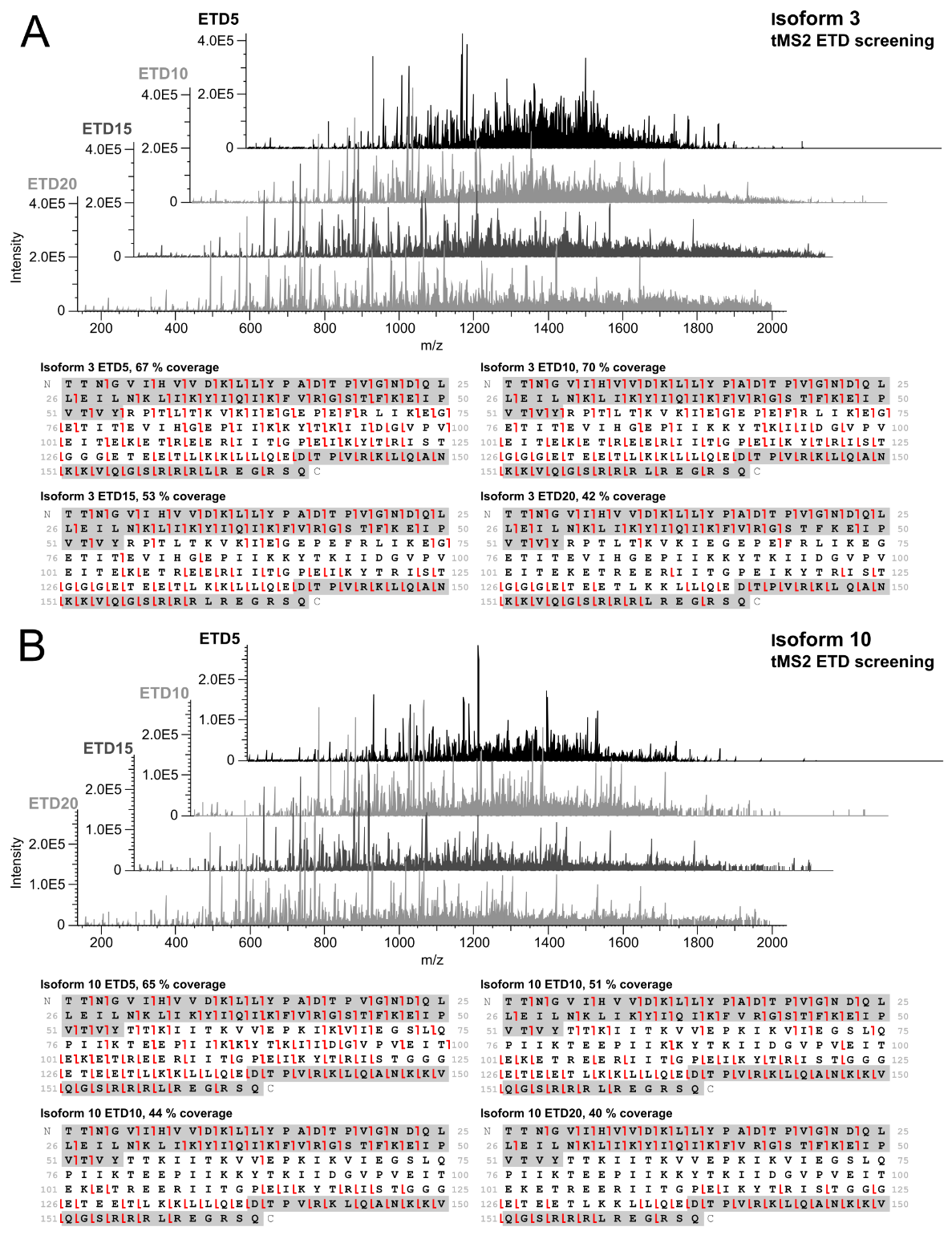
**

**Figure S5.** Isoforms 3 and 10 were subjected to tMS2 fragmentation screening of ETD with reaction times of 5, 10, 15, and 20 ms. A and B, MS/MS spectra (top) and fragmentation maps (bottom) from the different parameters are presented for isoform 3 (A) and isoform 10 (B). Fragmentation technique and sequence coverage is displayed above each fragmentation map. Fragments from the sequence highlighted in grey (c-ions in the C-terminal grey highlight, z-ions in the N-terminal grey highlight) are unique to the respective isoform. Red lines indicate c-/z-ions. Fragment tolerance is 10 ppm. For both isoforms, ETD5 and ETD10 outperform the other fragmentation techniques in terms of sequence coverage and distribution of fragments across the sequence.

| **Isoform or fragment** | **m/z values and z used in tMS2 fragmentation screening** | | **m/z values and z used in PRM method** | | **m/z values and z used in multiplexing method** | |
| --- | --- | --- | --- | --- | --- | --- |
|  | **m/z value** | **z** | **m/z value** | **z** | **m/z value** | **z** |
| **Isoform 1** | 834.6 | 30 | 782.5 | 32 | 834.6 | 30 |
|  |  |  |  |  | 782.5 | 32 |
|  |  |  |  |  | 736.6 | 34 |
| **Isoform 2** | 851.7 | 22 | 720.8 | 26 | 936.8 | 20 |
|  |  |  |  |  | 814.8 | 23 |
|  |  |  |  |  | 694.2 | 27 |
| **Isoform 3** | 790.6 | 24 | 824.9 | 23 | 948.5 | 20 |
|  |  |  |  |  | 903.4 | 21 |
|  |  |  |  |  | 824.9 | 23 |
| **Isoform 4** | 864.7 | 18 | 915.5 | 17 | 915.5 | 17 |
|  |  |  |  |  | 778.3 | 20 |
|  |  |  |  |  | 707.6 | 22 |
| **Isoform 5** | 738.3 | 30 | 738.3 | 30 | 763.7 | 29 |
|  |  |  |  |  | 738.3 | 30 |
|  |  |  |  |  | 651.6 | 34 |
| **Isoform 6** | 846.7 | 18 | 846.7 | 18 | 896.5 | 17 |
|  |  |  |  |  | 846.7 | 18 |
|  |  |  |  |  | 802.2 | 19 |
| **Isoform 7** | 804.5 | 15 | 710.0 | 17 | 1340.2 | 9 |
|  |  |  |  |  | 1096.7 | 11 |
|  |  |  |  |  | 710.0 | 17 |
| **Isoform 8** | 781.0 | 28 | 874.5 | 25 | 874.5 | 25 |
|  |  |  |  |  | 840.9 | 26 |
|  |  |  |  |  | 809.8 | 27 |
| **Isoform 9** | 749.5 | 29 | 869.2 | 25 | 869.2 | 25 |
|  |  |  |  |  | 776.2 | 28 |
|  |  |  |  |  | 724.5 | 30 |
| **Isoform 10** | 773.3 | 24 | 773.3 | 24 | 883.7 | 21 |
|  |  |  |  |  | 843.5 | 22 |
|  |  |  |  |  | 773.3 | 24 |
| **Shared fragment (Met 🡪 Hsl)** | Not included | | 1133.2 | 7 | 1133.2 | 7 |
|  |  |  |  |  | 991.7 | 8 |
|  |  |  |  |  | 881.6 | 9 |

**Table S1**. The precursor m/z value and charge state (z) used for targeted MS/MS are listed for the indicated experiments. The observed m/z value from the MS1 experiment is used in the targeted experiments. Hsl; homoserine lactone.

| **MS1 experiment in which the shared fragment is analyzed** | **Observed mass, monoisotopic** | **Theoretical mass, monoisotopic** | **Mass deviation (ppm)** | **Observed charge states** |
| --- | --- | --- | --- | --- |
| **MS1 of isoform 1** | 7921.33 | 7921.31 | 2.1 | 4-12 |
| **MS1 of isoform 2** | 7921.32 |  | 1.9 | 4-11 |
| **MS1 of isoform 3** | 7921.31 |  | 0.6 | 4-12 |
| **MS1 of isoform 4** | 7921.31 |  | 0.8 | 4-12 |
| **MS1 of isoform 5** | 7921.35 |  | 5.5 | 5-11 |
| **MS1 of isoform 6** | 7921.32 |  | 1.4 | 5-12 |
| **MS1 of isoform 7** | 7921.32 |  | 1.4 | 4-12 |
| **MS1 of isoform 8** | 7921.31 |  | 0.8 | 4-12 |
| **MS1 of isoform 9** | 7921.32 |  | 1.8 | 4-12 |
| **MS1 of isoform 10** | 7921.32 |  | 1.0 | 4-12 |

**Table S2.** The shared fragment (_545_TSEE…SDIM_615_) is produced by CNBr cleavage of all isoforms. Each row presents the observed monoisotopic mass of the shared fragment, mass deviation to the theoretical mass, and observed charge states for the shared fragment from the MS1 experiment of the respective isoforms indicated in the left column. The displayed mass is the shared fragment with its C-terminal methionine residue modified to a homoserine lactone (-48.00337 Da), which takes place following the CNBr reaction.

| **Charge** | **Isoform 1** | **Isoform 2** | **Isoform 3** | **Isoform 4** | **Isoform 5** | **Isoform 6** | **Isoform 7** | **Isoform 8** | **Isoform 9** | **Isoform 10** | **Shared fragment (Met 🡪 Hsl)** |
| --- | --- | --- | --- | --- | --- | --- | --- | --- | --- | --- | --- |
| **4** |  |  |  |  |  |  |  |  |  |  | 1982.34 |
| **5** |  |  |  |  |  |  |  |  |  |  | 1586.074 |
| **6** |  |  |  |  |  |  |  |  |  |  | 1321.896 |
| **7** |  |  |  |  |  |  |  |  |  |  | 1133.197 |
| **8** |  |  |  |  |  |  | 1507.593 |  |  |  | 991.6733 |
| **9** |  |  |  | 1728.308 |  |  | 1340.195 |  |  |  | 881.5994 |
| **10** |  |  |  | 1555.579 |  |  | 1206.276 |  |  |  | 793.5403 |
| **11** |  |  |  | 1414.254 |  | 1384.954 | 1096.706 |  |  |  | 721.4918 |
| **12** |  |  |  | 1296.483 |  | 1269.62 | 1005.398 |  |  |  | 661.4518 |
| **13** |  | 1440.731 | 1458.666 | 1196.831 |  | 1172.034 | 928.1367 |  |  |  |  |
| **14** |  | 1337.892 | 1354.546 | 1111.415 |  | 1088.389 | 861.9133 |  |  | 1324.971 |  |
| **15** |  | 1248.768 | 1264.31 | 1037.388 |  | 1015.898 | 804.5197 |  |  | 1236.706 |  |
| **16** |  | 1170.782 | 1185.354 | 972.6142 |  | 952.467 | 754.3002 |  |  | 1159.474 |  |
| **17** |  | 1101.972 | 1115.686 | 915.4602 |  | 896.4983 | 709.9889 |  |  | 1091.329 |  |
| **18** |  | 1040.807 | 1053.759 | 864.6574 |  | 846.7489 | 670.6011 | 1214.246 |  | 1030.756 |  |
| **19** |  | 986.0803 | 998.3506 | 819.2021 |  | 802.2366 | 635.3592 | 1150.392 |  | 976.5581 |  |
| **20** |  | 936.8264 | 948.4838 | 778.2924 |  | 762.1752 | 603.6414 | 1092.922 | 1086.311 | 927.7805 |  |
| **21** |  | 892.2634 | 903.3653 | 741.2789 |  | 725.9295 |  | 1040.926 | 1034.631 | 883.6484 |  |
| **22** | 1137.778 | 851.7519 | 862.3494 | 707.6304 |  | 692.9785 |  |  | 987.6497 | 843.5281 |  |
| **23** |  | 814.763 | 824.8999 | 676.9075 |  | 662.8921 |  | 950.498 | 944.7518 | 806.8971 |  |
| **24** | 1043.045 | 780.8569 | 790.5712 | 648.7452 |  | 635.3133 |  | 910.9359 | 905.427 | 773.3181 |  |
| **25** |  | 749.6628 | 758.9887 | 622.8356 | 885.8177 | 609.9406 |  | 874.5386 | 869.2507 | 742.4261 |  |
| **26** | 962.8898 | 720.8686 | 729.8355 | 598.9191 |  |  |  | 840.9407 | 835.8585 | 713.9101 |  |
| **27** | 927.2675 | 694.2071 | 702.8419 |  | 820.2766 |  |  | 809.8324 | 804.9371 | 687.5061 |  |
| **28** | 894.1888 | 669.4499 | 677.7763 |  | 791.0162 |  |  | 780.946 | 776.2258 | 662.9884 |  |
| **29** | 863.3871 | 646.4002 | 654.4397 |  | 763.7751 |  |  | 754.0524 | 749.4941 | 640.1607 |  |
| **30** | 834.6426 | 624.8869 | 632.6583 |  | 738.3497 |  |  | 728.9505 | 724.5448 | 618.8555 |  |
| **31** | 807.7508 | 604.7611 | 612.2824 |  | 714.5659 |  |  | 705.468 | 701.2044 | 598.9254 |  |
| **32** | 782.5407 |  |  |  | 692.2652 |  |  | 683.4535 | 679.3223 |  |  |
| **33** | 758.8581 |  |  |  |  |  |  | 662.7737 | 658.7685 |  |  |
| **34** | 736.5679 |  |  |  | 651.5725 |  |  | 643.3098 | 639.4212 |  |  |
| **35** | 715.5523 |  |  |  |  |  |  | 624.9582 | 621.1805 |  |  |
| **36** |  |  |  |  |  |  |  |  |  |  |  |
| **37** | 676.9256 |  |  |  |  |  |  |  |  |  |  |
| **38** | 659.1376 |  |  |  |  |  |  |  |  |  |  |
| **39** | 642.2629 |  |  |  |  |  |  |  |  |  |  |
| **40** | 626.231 |  |  |  |  |  |  |  |  |  |  |

**Table S3.** All observed precursors for the periostin isoforms are listed in the table. The m/z value is the observed value from the MS1 experiment. The precursors targeted in the PRM and multiplexing method are marked with green and yellow, respectively. As an example, note that targeting of isoform 1 precursor with charge 33 (m/z of 758.86) would lead to co-isolation of isoform 3 precursor with charge 25 (m/z of 758.99), thus precursors has been carefully selected for the targeted assays. Hsl; homoserine lactone.

| **Isoform / fragment** | **Targeted m/z** | **Charge** | **The m/z closest to the targeted m/z** | **Isoform of which the closest m/z belongs** | **Difference to closest m/z value** |
| --- | --- | --- | --- | --- | --- |
| **Isoform1** | 782.5407 | 32 | 780.9495 | Iso 8 | 1.5912 |
| **Isoform2** | 720.8296 | 26 | 724.5137 | Iso 9 | -3.6841 |
| **Isoform3** | 824.903 | 23 | 820 | Iso 5 | 4.6645 |
| **Isoform4** | 915.4598 | 17 | 910.9401 | Iso 8 | 4.5197 |
| **Isoform5** | 738.3143 | 30 | 736.5679 | Iso 1 | 1.7464 |
| **Isoform6** | 846.7473 | 18 | 840.9447 | Iso 8 | 5.8026 |
| **Isoform7** | 709.9928 | 17 | 707.6302 | Iso 4 | 2.3626 |
| **Isoform8** | 874.5424 | 25 | 869.2135 | Iso 9 | 5.3289 |
| **Isoform9** | 869.2135 | 25 | 864.6571 | Iso 4 | 4.5564 |
| **Isoform10** | 773.3232 | 24 | 776.1913 | Iso 9 | -2.8681 |
| **Shared fragment (Met 🡪 Hsl)** | 1133.19714 | 7 | 1137.77805 | Iso1 | -4.58091 |

**Table S4.** This table shows the m/z value of the precursors targeted in the PRM assay and their closest m/z value from another isoform. All targeted precursors have > 1.59 m/z value to the closest m/z value from another isoform. An isolation window of 1.2 m/z is used to avoid co-isolation. Hsl; homoserine lactone.
